# *Neisseria gonorrhoeae* and *Chlamydia trachomatis* infection in HIV-1-infected women taking antiretroviral therapy: a prospective cohort study from Burkina Faso

**DOI:** 10.1136/sextrans-2013-051233

**Published:** 2013-12-13

**Authors:** Andrea J Low, Issouf Konate, Nicolas Nagot, Helen A Weiss, David Mabey, Michel Segondy, Peter Vickerman, Nicolas Meda, Philippe van de Perre, Philippe Mayaud, for the Yerelon Cohort study group

**Affiliations:** 1 Department of Clinical Research, London School of Hygiene and Tropical Medicine, London, UK; 2 Unité de Recherche Santé de la Reproduction, VIH et Maladies Associées, Centre Muraz, Bobo-Dioulasso, Burkina Faso; 3 Departement de l'Information Medicale et Bacterologie-Virologie, INSERM U1058 Universite Montpellier-1 & CHU Montpellier, Montpellier, France

**Keywords:** Africa, Anteretroviral Therapy, Chlamydia Infection, Gonorrhoea, HIV

## Abstract

**Objectives:**

*Neisseria gonorrhoeae* (NG) and *Chlamydia trachomatis* (CT) are common sexually transmitted infections (STI). We assessed the cumulative risk of NG and CT in a cohort of HIV-1-infected high-risk women taking antiretrovirals over 4 years in Burkina Faso.

**Methods:**

Between March 2007 and February 2011, participants were followed every 3–6 months. At each visit, participants underwent a gynaecological examination with collection of cervical and vaginal swabs. Random-effects logistic regression models were used to analyse associations of NG and CT infection with behavioural and biological factors.

**Results:**

172 women had samples tested for NG and CT during the study period, in a total of 1135 visits. NG was detected in 6.4% of women (11/172, 95% CI 2.7 to 10.1) at a rate of 2.76 cases (95% CI 1.53 to 4.99) per 100 person-years. CT was detected in 1.7% (3/172, 95% CI 0 to 3.7) of women at a rate of 0.75 cases (95% CI 0.24 to 2.34) per 100 person-years. The majority of women were asymptomatic (9/14). In the multivariable model, the presence of NG or CT was associated with tobacco use (aOR=11.85, 95% CI 1.13 to 124.17), and concurrent genital HIV-1 RNA shedding (aOR=4.78, 95% CI 1.17 to 19.46). Higher levels of education (aOR=0.17, 95% CI 0.03 to 0.92), and age greater than 35 years (aOR=0.07, 95% CI 0.01 to 0.92) were associated with lower odds of infection.

**Conclusions:**

The risk of NG or CT infection remains low among high-risk women in Bobo-Dioulasso. This provides some evidence that antiretroviral use does not contribute to behavioural disinhibition. The asymptomatic nature of most infections underscores the need for regular screening and treatment of STIs in core groups.

## Introduction

*Neisseria gonorrhoeae* (NG) and *Chlamydia trachomatis* (CT) are two of the most common bacterial sexually transmitted infections (STIs). Consequences of infection include infertility and an increased risk of HIV transmission. Syndromic management of STIs was adopted in Burkina Faso in 1996, corresponding to a decline in the prevalence of bacterial STIs.^1^ There is concern that sexual behaviour disinhibition, or lowered rates of protected sexual intercourse might occur in HIV-infected individuals with improving access to antiretrovirals (ART).^2^ As female sex workers (FSW) are an important core group for the transmission of STIs and HIV in West Africa, we assessed the cumulative risk of NG and CT in a cohort of HIV-1-infected high-risk women taking ART over 4 years in Burkina Faso.

## Methods

Study participants were HIV-1-seropositive women from the Yerelon cohort living in Bobo-Dioulasso, Burkina Faso. This cohort was established in 1998 to assess the impact of interventions in reducing HIV acquisition among high-risk women. Women working in the streets and bars of Bobo-Dioulasso were eligible for the cohort if they reported at least one transactional sex act per week, were aged 16 years or older, and were willing to undergo regular testing for HIV and STIs. Women were also recruited from local organisations for people living with HIV/AIDS, using the same criteria.^3^ Since 2004, HIV-infected participants who meet the WHO eligibility criteria for ART initiation have had access to ART. We restricted this analysis to visits after 2007 due to concerns about genital sample degradation, and to participants who took ART during the study period, and included the baseline visit for those initiating ART.

Between March 2007 and February 2011, participants were followed every 3–6 months. At each study visit, a full behavioural and clinical questionnaire was administered, and a gynaecological exam conducted. Samples from unscheduled intervening visits were not tested. Cervical swabs were collected and dry-stored at −80°C. Vaginal swabs were also collected and examined for *Trichomonas vaginalis* (TV), bacterial vaginosis (BV), and *Candida albicans* (CA) using microscopy. Diagnosis of NG and CT was done posthoc on cervical swabs using PCR (Amplicor CT/NG PCR assay, Roche) using a pooling approach. An enriched cervico-vaginal lavage (eCVL) was performed and tested for the presence of HIV-1 RNA and Herpes Simplex Virus Type 2 (HSV-2) DNA (in HSV-2 seropositive women) using real-time PCR, and semen using Y-PCR, as previously described.^4^

Statistical analysis was conducted using Stata 12.0. Cumulative risk was calculated as the number of new cases of NG or CT during the study period divided by the number of women at risk, and excluded any cases at baseline. To determine factors associated with detection of NG or CT, ORs were estimated using random-effects logistic regression, adjusting for repeated measures and within-woman correlation. p Values were calculated using likelihood-ratio tests. A multivariable model was constructed using a conceptual hierarchical framework, including factors either decided a priori*,* namely reported number of sex acts in the past week, or independently associated with the presence of NG or CT in the univariable model, using p≤0.10 as the inclusion criterion. The research protocol was approved by the research ethics committees at the London School of Hygiene and Tropical Medicine and the Burkina Faso Ministry of Health. All women provided written informed consent.

## Results

During the study period, there were 305 women in the cohort, 258 of whom were HIV-1 infected, and 180 who were on ART. Cervical swab samples for NG/CT testing were available from 172 (96%) of the women taking ART. At the first visit with NG/CT results, the mean time of their participation in the cohort was 3.1 years (±SD: 1.4), the median age was 32.5 years (range 18–50), and the mean CD4 count was 366 cells/μL (±SD: 211). Seventy per cent (120/172) were already taking ART at the first visit (mean duration on ART 2.0 years (±SD: 1.3)). There was one case of NG at baseline, which was excluded from further analyses.

The median length of follow-up was 34.7 months with a median number of visits of 4 (range 1–10), for a total of 1135 visits. The mean number of sex acts in the past week was 3.2 (SD ±8.9). In visits with recorded data, women reported using condoms often or always in 73% (795/1095) of visits, and reported receiving STI syndromic management in the past month in 18% (173/972) of visits. During follow-up, NG was detected in 6.4% of women (11/172, 95% CIs: 2.7 to 10.1) at a rate of 2.76 cases (95% CI 1.53 to 4.99) per 100 person-years (py). CT was detected in 1.7% (3/172, 95% CI 0 to 3.7) of women at a rate of 0.75 cases (95% CI 0.24 to 2.34) per 100 py, for a total of 1.2% (14/1135, 95% CI 1.0 to 1.9) of visits. There were no cases of co-infection. Semen was detected in 9% (79/873), TV in 1.2% (13/1065), BV in 36.6% (386/1054), and CA in 5.5% (59/1070) of tested samples. The most common presentation was asymptomatic (n=9); symptoms were genital pruritus (n=2), dysuria (n=2), pelvic pain (n=2), and dyspareunia (n=1). Women were seen in 2.8% (54/1963) of unscheduled visits for symptoms of a possible STI, although these were not confirmed with laboratory testing. Relevant clinical findings were abnormal vaginal discharge (n=1) and an abnormal cervical exam (n=3). In the multivariable model, the presence of NG or CT was associated with tobacco use (adjusted OR (aOR)=11.85, 95% CI 1.13 to 124.17), and concurrent genital HIV-1 RNA shedding (aOR=4.78, 95% CI 1.17 to 19.46), and weakly associated with injectable contraceptive use (depot medroxyprogesterone acetate or DMPA) (aOR=5.83, 95% CI 0.90 to 37.70) (table 1). Higher levels of education (aOR=0.17, 95% CI 0.03 to 0.92), and age greater than 35 years (aOR=0.07, 95% CI 0.01 to 0.92) were associated with lower odds of infection. The number of recent sex acts, reported condom use, physical findings, alcohol use, other STIs and the presence of HSV-2 DNA or semen were not associated with NG/CT detection.

**Table 1:** Univariable and multivariable analysis of cumulative risk of *Neisseria gonorrhoeae* and *Chlamydia trachomatis* in 172 high-risk women in Burkina Faso

Characteristics*	Visits with NG or CT n/N (%) (N=1135)	Unadjusted OR† (95% CI)	p Value‡	Adjusted OR (95% CI)	p Value‡
Age groups, years					
18–24	4/145 (2.8)	1.0	0.06	1.0	0.02
25–34	9/541 (1.7)	0.59 (0.18 to 1.98)		0.99 (0.19 to 5.28)	
≥35	1/445 (0.2)	0.08 (0.01 to 0.72)		0.07 (0.01 to 0.92)	
Education					
None	10/522 (1.9)	1.0	0.03	1.0	0.02
Primary/above	4/609 (0.7)	0.34 (0.10 to 1.10)		0.17 (0.03 to 0.92)	
Regular tobacco use					
No	12/1102 (1.1)	1.0	0.08	1.0	0.05
Yes	2/33 (6.1)	5.96 (1.19 to 29.87)		11.85 (1.13 to 124.17)	
Hormonal contraceptive use					
None	11/937 (1.2)	1.0	0.06	1.0	0.04
Oral contraceptives	0/99 (0)	NE		NE	
DMPA	3/39 (7.7)	7.02 (1.87 to 26.25)		5.83 (0.90 to 37.70)	
Number of sex acts in past week					
0	2/393 (0.5)	1.0	0.12	1.0	0.34
1–4	6/373 (1.3)	3.23 (0.55 to 19.10)		2.55 (0.43 to 14.91)	
≥5	3/120 (2.5)	5.67 (0.81 to 39.77)		3.93 (0.54 to 28.78)	
Concurrent CD4 count					
≥100 cells/μL	12/1101 (1.1)	1.0	0.02	1.0	0.29
<100 cells/μL	2/16 (12.5)	14.20 (2.36 to 85.38)		4.54 (0.36 to 57.00)	
eCVL HIV-1 RNA detected					
No	7/791 (0.9)	1.0	0.06	1.0	0.03
Yes	7/276 (2.5)	2.92 (1.00 to 8.52)		4.78 (1.17 to 19.46)	

*Denominators vary due to missing data.

†ORs determined by random effects logistic regression.

‡p Values calculated using likelihood ratio tests; Factors not associated with infection with NG or CT in univariable analysis (and therefore not presented) were regular alcohol use, full-time sex work, reported condom use, regular vaginal washing, antibiotic use in the past month, abnormal vaginal discharge on exam, genital ulcers or vesicles on exam, abnormal cervical exam, genital warts, concurrent bacterial vaginosis, *Trichomonas vaginalis*, *Candida albicans*, or HSV-2 DNA, presence of Y-PCR, HIV-1 plasma viral load, time since sample collection, or antiretroviral status.

CT, *Chlamydia trachomatis*; DMPA, depot medroxyprogesterone acetate; eCVL, enriched cervico-vaginal lavage; NE, not estimable; NG, *Neisseria gonorrhoeae*.

## Discussion

The adoption of STI syndromic management in 1996 in Bobo-Dioulasso occurred concurrently with a national programme increasing condom availability. By the year 2000, the prevalence of bacterial STIs in the general population had decreased, while HIV prevalence had stabilised.^1^ Among FSWs in Bobo-Dioulasso, there was a decline in NG prevalence from 13% in 1994 to 1% in 2000, and of CT prevalence from 10% in 1994 to 2.7%. Our findings suggest that this impact has been maintained over a longer period. Moreover, the data do not support the notion of a widespread risk of migration out of condom use among high-risk women taking ART. However, caution must be exercised in comparing this data with prior surveys, as we are measuring cumulative risk and not prevalence, and as the women in our study are HIV positive and are older, and this data might reflect reduced sexual activity. Furthermore, the women had already been followed in the cohort for a mean duration of 3 years, and had optimal access to condoms and to care. It is likely that the rate of infection is an underestimate, as we did not test interim samples and women reported significant use of syndromic treatment. The use of older samples might have reduced the sensitivity of our test, but there was no association between detection and time since sample collection. Thus, our data might reflect a combination of reduced exposure through reduced sexual activity, not capturing infections, improved adherence with safer sex practices, and possibly a lower prevalence of STIs among the sexual partners of these women.

In this study, NG/CT was associated with tobacco and weakly with DMPA use, and HIV-1 RNA shedding. Tobacco has been linked to CT infection in other studies,^5^ potentially due to biological effects on the cervical mucosa, such as depressing the production of inflammatory cytokines.^6^ However, tobacco use has also been strongly linked to high-risk sexual behaviour.^5^ DMPA has been associated with an increased risk of CT infection in a study of HIV-infected FSWs in Kenya.^7^ Studies of macaques have shown that DMPA causes cervico-vaginal thinning, although the effect of DMPA varies in humans.^8^ Progesterone also impairs NK cell function and the migration of lymphocytes and macrophages to the genital tract, resulting in diminished cellular immune responses. A meta-analysis examining this association found highly varied results, and inadequate controlling for sexual behaviour in most studies.^9^ As we relied on self-report to measure sexual behaviour, there is a strong possibility of unmeasured high-risk activities and residual confounding. This is also likely reflected in the protective effect seen with older age and higher educational level. However, the small number of infections and women on DMPA make this association weak and, thus, difficult to interpret.

The association between NG/CT infection and HIV-1 RNA shedding is consistent with studies of ART naive women and is a likely reflection of a proinflammatory effect of NG/CT infection on the cervico-vaginal mucosa, potentially driving local replication of HIV-1, and not a causal effect of HIV-1 shedding on NG/CT acquisition.^10^

Finally, the large number of women who were asymptomatic on presentation, and the low sensitivity for clinical findings for NG/CT infection demonstrates the need for regular screening and treatment of STIs in core groups. However, it is reassuring that, in a potentially high-risk group, we are not identifying more cases of NG/CT, particularly with the global rise in antibiotic resistance and the potential for behavioural disinhibition while on ART.

## Supplementary Material

Web

## References

[1] Nagot N Meda N Ouangre A Review of STI and HIV epidemiological data from 1990 to 2001 in urban Burkina Faso: implications for STI and HIV control. Sex Transm Infect 2004;80:124–9 15054175 10.1136/sti.2002.004150 PMC1744820

[2] Behanzin L Diabate S Minani I Decline in the prevalence of HIV and sexually transmitted infections among female sex workers in Benin over 15 years of targeted interventions. J Acquir Immune Defic Syndr 2013;63:126–34 23337368 10.1097/QAI.0b013e318286b9d4 PMC3805545

[3] Nagot N Ouangre A Ouedraogo A Spectrum of commercial sex activity in Burkina Faso: classification model and risk of exposure to HIV. J Acquir Immune Defic Syndr 2002;29:517–21 11981369 10.1097/00126334-200204150-00013

[4] Nagot N Foulongne V Becquart P Longitudinal assessment of HIV-1 and HSV-2 shedding in the genital tract of West African women. J Acquir Immune Defic Syndr 2005;39:632–4 16044019

[5] Wolf R Freedman D Cigarette smoking, sexually transmitted diseases, and HIV/AIDS. Int J Derm 2000;39:1–9 10651955 10.1046/j.1365-4362.2000.00721.x

[6] Lieberman JA Moscicki AB Sumerel JL Determination of cytokine protein levels in cervical mucus samples from young women by a multiplex immunoassay method and assessment of correlates. Clin Vaccine Immunol 2008;15: 49–54 17978011 10.1128/CVI.00216-07 PMC2223863

[7] Lavreys L Chohan V Overbaugh J Hormonal contraception and risk of cervical infections among HIV-1-seropositive Kenyan women. AIDS 2004;18:2179–84 15577651 10.1097/00002030-200411050-00010

[8] Kaushic C Roth KL Anipindi V Increased prevalence of sexually transmitted viral infections in women: the role of female sex hormones in regulating susceptibility and immune responses. J Reprod Immunol 2011;88:204–9 21296427 10.1016/j.jri.2010.12.004

[9] Mohllajee AP Curtis KM Martins SL Hormonal contraceptive use and risk of sexually transmitted infections: a systematic review. Contraception 2006;73:154–65 16413846 10.1016/j.contraception.2005.08.012

[10] Johnson LF Lewis DA The effect of genital tract infections on HIV-1 shedding in the genital tract: a systematic review and meta-analysis. Sex Transm Dis 2008;35:946–59 18685546 10.1097/OLQ.0b013e3181812d15

