# Supplementary material for: Neisseria gonorrhoeae and Chlamydia trachomatis infection in HIV-1-infected women taking antiretroviral therapy: a prospective cohort study from Burkina Faso
Source: Sex Transm Infect. 2013 Dec 13;90(2):100–3. doi: 10.1136/sextrans-2013-051233 (PMC3932980; doi:10.1136/sextrans-2013-051233)
Supplement: Web [file sextrans-2013-051233-s1.pdf]

Objectifs: *Neisseria gonorrhoeae* (NG) et *Chlamydia trachomatis* (CT) sont des infections sexuellement transmissibles communs (IST). Nous avons évalué le risque cumulatif de NG et CT dans une cohorte de femmes infectées par le VIH-1 à haut risque qui prenaient des antirétroviraux durant 4 ans au Burkina Faso.

Méthodes : Entre Mars 2007 et Février 2011 les participantes ont été suivies tous les 3 à 6 mois. Lors de chaque visite, les participants ont subi un examen gynécologique avec collection d'échantillons cervicaux et vaginaux. Des modèles de régression logistiques à effets aléatoires ont été utilisés pour analyser les associations d'infection NG et CT avec des facteurs comportementaux et biologiques.

Résultats : 172 femmes avaient des échantillons testés pour NG et CT au cours de la période d'étude, pour un total de 1135 visites. NG a été détectée dans 6,4% des femmes (11/172, IC à 95 % : 2,7 à 10,1) à un taux de 2,76 cas (IC à 95%:1,53 - 4,99) pour 100 années-personnes. CT a été détectée dans 1,7% (3/172, IC à 95%: 0-3,70) des femmes à un taux de 0,75 cas (IC à 95%: 0,24 à 2,34) pour 100 années-personnes. La majorité des femmes étaient asymptomatiques (9/14). Dans le modèle multivarié, la présence de NG ou CT a été associé à la consommation de tabac (ORa = 11,85, IC à 95%:1,13 -124,17), et l'excrétion génitale du VIH-1 ARN (ORa = 4,78, IC à 95%:1,17 -19,46). Des niveaux plus élevés de l'éducation (ORa = 0,17, IC à 95%:0,03 -0,92) et un âge supérieur à 35 ans (ORa = 0,07, IC à 95%:0,01 -0,92) étaient associés à un risque plus faible d'infection.

Conclusions : Le risque d'infection avec NG ou CT demeure faible chez les femmes à haut risque de Bobo- Dioulasso. Ceci donne une certaine preuve que l'utilisation des antirétroviraux ne contribue pas à la désinhibition comportementale. La nature asymptomatique de la plupart des infections souligne la nécessité d'un dépistage et traitement des IST régulier dans les groupes à haut-risque.

BMJ Publishing Group Limited takes no responsibility for the accuracy of the translation from the published English original and is not liable for any errors that may occur.
